# Editorial

**Published:** 2013-09-06

**Authors:** Shibal Bhartiya, Tarek Shaarawy, Tanuj Dada

I do not pretend we have all the answers. But the questions are certainly worth thinking about.

―Arthur C Clarke

The editorial team of the Journal of Current Glaucoma Practice takes great pleasure in bringing to you this issue of the Journal. We hope you will find this issue insightful and relevant to your practice of glaucoma.

The editorial team of the Journal of Current Glaucoma Practice (JOCGP) hopes you will find the current issue insightful and relevant to your practice of glaucoma.

Pinto et al determine the morphometric parameters of filtration blebs using magnetic resonance imaging (MRI) scans following Ahmed valve implantation and report that bleb morphology seems to correlate with both the pre- and postoperative IOP, which might suggest a clinical benefit of administering aqueous suppressants pre- as well as postoperatively. They also report that the plate of the device may show a significant dislocation from its initial surgical implantation site.

Gil-Carrasco et al evaluate the ability of phacoemulsification combined with either primary trabeculectomy (PT) or primary Ahmed glaucoma valve implantation (PAVI) to achieve target intraocular pressures in adults with primary open angle glaucoma. They report that midterm results for achieving target pressures using combined phacoemulsification with either PT or PAVI are comparable. The profile of complications is different for the two procedures.

Complications of glaucoma surgery can be sight-threatening, and vision loss does occur more frequently in eyes with chronic hypotony following glaucoma surgery. However, Yun et al report that not all eyes with chronic hypotony develop sight-threatening complications and propose that the definition of hypotony should combine IOP criteria with the presence of structural and/or functional changes.

Bhartiya et al investigated the diurnal IOP fluctuations in eyes with various subtypes of angle-closure disease, and found that the mean fluctuation in IOP was found to significantly increase with increase in disease severity. The authors also report a significant difference between the mean diurnal IOP and peak diurnal IOP between the three groups, with a strong correlation between peak IOP and fluctuation.

Kanadani et al report that, although the systemic hypertensive patients have a higher ocular perfusion pressure in comparison to normal patients, this increase does not mean that they also have a higher ocular blood flow (as measured by POBF tonograph). This may be caused by chronic changes in the vascular network and in the blood hemodynamics in patients with systemic hypertension.

Iatrogenic pigment dispersion syndrome generally originates from a repetitive, mechanical trauma to the pigmented posterior epithelium of the iris. Van Mierlo et al report that IOL-exchange appears to be a useful tool in the management of iatrogenic pigment dispersion glaucoma due to inappropriate IOL implantation in this subgroup of patients. They suggest that this cause-oriented approach may be effective in controlling IOP, but should be offered only if safety criteria are met.

Ichhpujani et al review the current status of collagen implants in glaucoma surgery, concluding that the use of the biodegradable implants in glaucoma surgery is still evolving, and further studies are needed to find the appropriate surgical technique, the ideal size and site of placement and determine their long-term impact on trabeculectomy outcomes and complications.

As always, we look forward to hearing from you.

Best wishes

**Shibal Bhartiya**

**Tarek Shaarawy**

**Tanuj Dada**

